# Plasma metabolic profiling reveals exploratory metabolic differences in chronic coronary syndrome with coronary artery calcification

**DOI:** 10.3389/fcvm.2026.1741496

**Published:** 2026-06-26

**Authors:** Xiaoxue Ding, Haiyan Wu, Mingjie Pang, Shiqi Liu, Haoqiang Chen, Ke Zhuang, Wenhua Su, Yan Zhao, Hong Zhang

**Affiliations:** 1 Department of Cardiovascular Medicine, The First People's Hospital of Yunnan Province, Kunming, Yunnan, China; 2 Department of Cardiovascular Medicine, The Affiliated Hospital of Kunming University of Science and Technology, Kunming, Yunnan, China; 3 Faculty of Life Science and Biotechnology, Kunming University of Science and Technology, Kunming, Yunnan, China

**Keywords:** arachidonic acid, chronic coronary syndrome, coronary artery calcification, metabolomics, oleic acid

## Abstract

**Background/objectives:**

Coronary artery calcification (CAC) is a specific marker of coronary atherosclerotic heart disease, with chronic coronary syndrome (CCS) being a primary clinical manifestation of coronary artery disease. However, research on the plasma metabolites of individuals with CCS and CAC, as well as the identification of candidate markers is scarce. Here we aimed to explore metabolic differences among CAC patients, CCS patients, and healthy controls.

**Methods:**

We conducted a study involving 30 patients with CCS (CCS group), 30 with CAC (CAC group), and 30 healthy controls (Control group). Plasma parameters were statistically analyzed. Moreover, metabolomics analysis explored the composition and functions of metabolites in the plasma of each group.

**Results:**

We found that serum creatinine and fasting glucose levels were significantly increased in the CAC group, whereas blood urea nitrogen levels were significantly higher in both the CAC and CCS groups compared with Control group. Significant metabolic differences were observed between the CCS and CAC groups. Further analyses indicated that oleic acid and arachidonic acid were among the metabolites contributing to the separation between CAC patients and healthy controls.

**Conclusion:**

These findings suggest that plasma metabolite profiles differ among the study groups. The identified metabolites may contribute to distinguishing CAC patients from those with CCS as well as healthy controls, providing additional insights into the metabolic alterations associated with these conditions.

## 1 Introduction

Although the prevalence and related mortality of cardiovascular diseases have declined globally in the past few decades (1), these conditions remain to be the leading causes of mortality and morbidity in many countries. Chronic coronary syndrome (CCS) is a clinical manifestation of coronary artery disease (2) and drives the progression of chronic diseases (3). The main goal in treating CCS patients is to reduce the risk of adverse cardiac events, including death (4). Studies have shown that changing lifestyle and optimizing drug treatment can significantly reduce the impact of risk factors (5–7). Coronary artery calcification (CAC) is a common feature observed in CCS patients, which is characterized by decreased vascular compliance, abnormal vasomotor response, and reduced myocardial perfusion (8–11). CAC accelerates the progression of cardiovascular diseases (12, 13). Its severity is directly related to the severity of atherosclerosis and is considered to be a marker of cardiovascular adverse reactions. CAC commonly occurs in advanced atherosclerosis 14 and is associated with the frequency of future cardiac events (15, 16).

To date, numerous biochemical markers and pathways have been shown to be contributors to calcification. Elevated Lp(a) levels have been associated with higher CAC volume, especially in individuals with elevated markers of inflammation and coagulation (17). Current studies have found that serum irisin, sclerostin, hypoxia inducible factor-1α, fetuin A and other factors are closely related to calcification (18–21), which therefore are expected to become non-invasive serological indicators for the early detection and monitoring of vascular calcification. Guo et al. analyzed the clinical characteristics of CAC in maintenance hemodialysis patients, identifying serum irisin and sclerostin as potential indicators for the diagnosis and evaluation of CAC severity (22). Moreover, studies have shown that early interventions in cardiovascular diseases can delay progression, improve life quality, and reduce medical cost of patients (23, 24). Therefore, identifying specific metabolic features associated with vascular calcification may help improve early detection, prevention, and management of CAC.

In this study, Ultra-High Performance Liquid Chromatography coupled with Q Exactive Mass Spectrometer (UHPLC-QE-MS) was used to explore the plasma metabolites of 30 patients with CAC, 30 patients with CCS but without CAC, and 30 healthy controls. Bioinformatics analyses, including weighted gene co-expression network analysis (WGCNA) and random forest modeling, were performed to explore metabolites associated with differences between the CAC and CCS groups. Our findings provide preliminary insights into the metabolic alterations associated with CAC and CCS, which may inform future studies on cardiovascular disease mechanisms and potential early detection strategies.

## 2 Material and methods

### 2.1 Study design

From November 2021 to January 2022, we consecutively recruited patients who had received treatment in the Cardiovascular Department at the First People's Hospital of Yunnan, China. Coronary angiography was conducted on patients to assess for >50% stenosis in a single coronary. The diagnosis of coronary heart disease and CCS were made in compliance with ACC/AHA criteria (25). Based on coronary angiography results, three groups were finally established: (i) patients with severe calcification who required rotational atherectomy and PCI with stenting (CAC group); Patients with no severe calcification and accepted PCI with stenting (CCS group); and healthy controls with normal coronary angiography (Control group). Exclusion criteria included malignancy, autoimmune disease, haematological disease or acute myocardial infarction within one month. All patients included in the study had no history of medication prior to their initial diagnosis at our hospital. All patients enrolled in the study were treatment-naive at the time of blood sampling, with no prior history of medication, including lipid-lowering therapy, before their initial diagnosis at our hospital. Key variables, including age, sex, smoking status, were compared between the study groups (Control vs. CAC, Control vs. CCS, and CAC vs. CCS) at enrollment, and no statistically significant differences were observed among the groups.

After fasting overnight, on the morning prior to the day of coronary angiography, blood samples were collected into heparinized tubes, and chilled to 4 °C for 2 h. Plasma was isolated from the samples after centrifugation at 3,000 rpm for 10 min, and then stored at −80 °C for examination.

The study was approved by the Ethics Committee of the First People's Hospital of Yunnan Province [2016LH083] and conformed to relevant ethical guidelines for human and animal research. It conformed to the principles outlined in the Declaration of Helsinki. Informed consent was obtained from all individuals included in this study.

### 2.2 Metabolite extraction

For each sample, 50 μL was transferred into an Eppendorf tube. After adding 200 μL of extract solution [acetonitrile:methanol = 1:1 (v/v), containing an isotopically-labelled internal standard mixture], samples were vortexed for 30 s, sonicated for 10 min in an ice-water bath, and then incubated at −40 ℃ for 1 h to precipitate proteins. The samples were then centrifuged at 12000rpm at 4 ℃ for 15 min. The resulting supernatants were transferred to fresh glass vials for metabolomic analysis. Quality control samples were prepared by mixing the equal aliquots of the supernatants obtained from all the samples.

### 2.3 UHPLC-QE-MS analysis

LC‒MS/MS analyses were performed using a UHPLC system (Vanquish, Thermo Fisher Scientific, MA, USA) with an Acquity UPLC BEH amide column (2.1 mm × 100 mm, 1.7 μm) coupled to a Q Exactive HFX mass spectrometer (Orbitrap MS, Thermo, MA, USA). The mobile phase consisted of 25 mmol/L ammonium acetate and 25 ammonium hydroxide in water (pH = 9.75) (A), and acetonitrile (B). The autosampler temperature was 4 ℃, and the injection volume was 3 μL. The QE HFX mass spectrometer was used to acquire MS/MS spectra in information-dependent acquisition (IDA) mode under the control of the acquisition software (Xcalibur, Thermo, MA, USA). In this mode, the acquisition software continuously evaluated the full-scan MS spectrum. The ESI source conditions were as follows: sheath gas flow rate was 30 Arb, Aux gas flow rate was 25 Arb, capillary temperature was 350 ℃, full MS resolution was 60,000, MS/MS resolution was 7,500, collision energy was 10/30/60 in NCE mode, and spray voltage was 3.6 kV (positive) or −3.2 kV (negative).

Raw data were converted into mzXML format using ProteoWizard, and subsequently processed using an in-house R package (based on XCMS) for peak detection, peak extraction, alignment, and integration. Metabolite annotation was then performed by matching MS/MS spectra against an in-house M2 database, BiotreeDB (v2.1), with a similarity score cutoff set at 0.3. All lipid species were putatively annotated (M2) based on MS/MS spectral matching. Due to the limitations of untargeted LC–MS/MS, detailed structural features such as double bond positions, geometric configurations, and sn-position or backbone isomerism could not be unambiguously resolved. Therefore, lipid species were reported at a level consistent with the analytical resolution, and a single annotation may correspond to multiple isomeric structures.

### 2.4 Bioinformatics and statistical analysis of metabolomic data

Raw data were converted to mzXML (version 2.38) format using ProteoWizard, and then processed for peak detection, extraction, alignment, and integration. The tool used for data processing was an in-house program that was developed using R package version 3.2 and was based on XCMS. An in-house MS2 database (BiotreeDB) was then applied for metabolite annotation. The cut-off for annotation was set at 0.3.

Principal component analysis (PCA) was conducted using the stats package, while orthogonal partial least-squares discriminant analysis (OPLS-DA) was performed using the ropls package in R (v. 4.4.1). Variable importance in projection (VIP) scores were calculated from the OPLS-DA model. Differential metabolites (DIMs) were defined as those with VIP > 1 and *p* < 0.05. These DIMs were subsequently mapped to the Kyoto Encyclopedia of Genes and Genomes (KEGG) database for pathway enrichment analysis using MetaboAnalyst 5.0 (26). Metabolite Set Enrichment Analysis (MSEA) was performed to identify significantly altered metabolic pathways. The top 10 pathways with the lowest *p*-values were selected for visualization in the bubble plot.

For statistical analysis, differences in demographic and clinical parameters between groups were evaluated using the Mann–Whitney U test, with *p* < 0.05 considered statistically significant. All statistical analyses were conducted in R software.

### 2.5 Construction of weighted co-expression network

Metabolites with low connectivity were filtered using the goodSamplesGenes function from the WGCNA package in R. Dynamic tree pruning was used to define significant co-expression modules, and a clustering dendrogram was constructed based on metabolite correlations. The minimum module size was set to 30 metabolites, and modules with similar eigengene profiles (correlation > 0.75) were merged. Module–trait correlations were calculated, with absolute correlation values closer to 1 indicating stronger association between module metabolites and the trait of interest. The metabolite co-expression network was visualized using Cytoscape (v3.10.0).

### 2.6 Metabolite correlation analysis

Quantitative metabolite levels across all samples were used to compute pairwise correlations. Pearson correlation coefficients were calculated using the cor function in R. DIMs with correlation coefficients > 0.65 and *p* < 0.05 were selected for downstream analysis.

### 2.7 Random forest modeling and receiver operating characteristic (ROC) curve analysis

A random forest algorithm was applied as an exploratory approach to prioritize metabolites that contributed to group differentiation. The dataset was randomly divided into training and test sets at a 7:3 ratio, and ten-fold cross-validation was performed for internal validation. The rfPermute package in R was used to perform 1,000 permutation tests and calculate *p*-values for the importance of each metabolite. The Mean Decrease Accuracy was used to quantify the contribution of each metabolite to the classification model, and only metabolites with *p* < 0.05 were retained.

ROC curve analysis was performed using the pROC package in R. The area under the curve (AUC) and its 95% confidence interval were calculated to assess the discriminatory power of each metabolite in distinguishing CAC and CCS patients from healthy controls, with higher AUC values indicating greater diagnostic potential.

## 3 Results

### 3.1 Baseline characteristics of the study population

**Figure 1:**
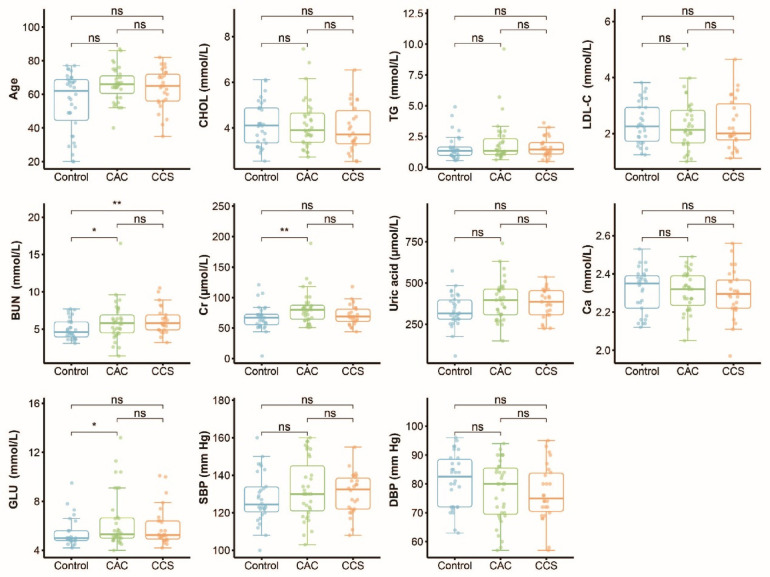
Boxplots showing baseline characteristics of participants. Differences between groups were assessed using the Mann–Whitney U test. Statistical significance is indicated as: * *p* < 0.05, ** *p* < 0.01, ns = not significant. CHOL, cholesterol; TG, triglycerides; LDL-C, low-density lipoprotein cholesterol; BUN, blood urea nitrogen; Cr, creatinine; Ca, calcium; GLU, fasting glucose; SBP, systolic blood pressure; DBP, diastolic blood pressure.

Blood parameters of the CAC, CCS, and Control groups, including diastolic blood pressure, systolic blood pressure (SBP), fasting glucose, calcium, and age, were analyzed. The results showed that creatinine and fasting glucose levels were significantly elevated only in the CAC group, whereas blood urea nitrogen levels were significantly higher in both the CAC and CCS groups compared with the Control group (Figure 1). No significant differences were observed for the other parameters.

### 3.2 Metabolomic profiling of CAC, CCS, and control groups

**Figure 2:**
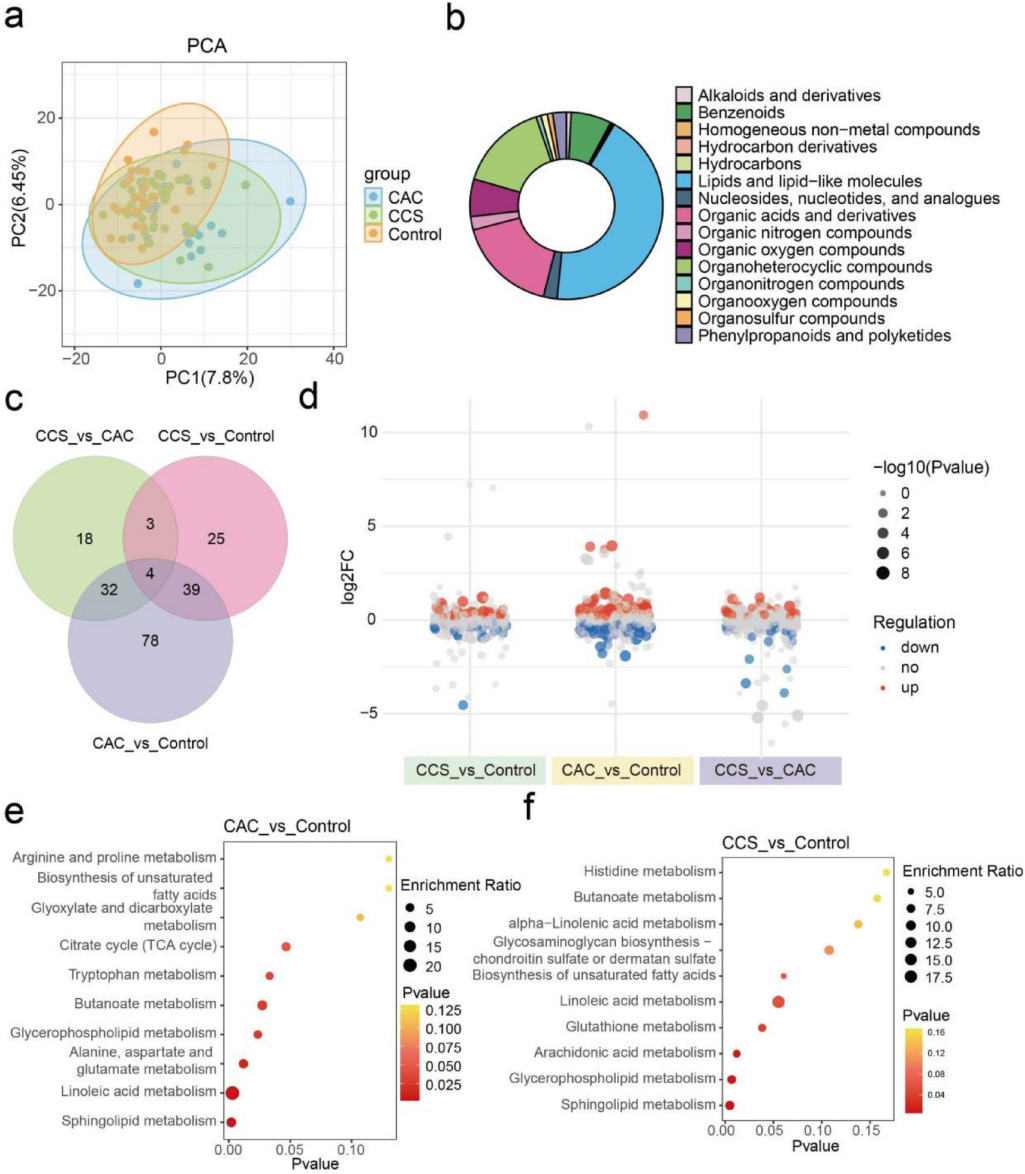
Metabolome differential analysis and KEGG functional enrichment analysis. **(a)** Principal component analysis (PCA) of all samples from CAC, CCS and Control groups. **(b)** Pie chart showing 15 classes of identified metabolites. **(c)** Venn Diagram demonstrating the number of overlapping and unique metabolites among CAC, and CCS and Control groups. **(d)** Volcano plot showing the distribution of DIMs among CAC, and CCS and Control groups. e-f: KEGG enrichment analysis of DIMs between CAC and Control groups **(e)**, as well as CCS and Control groups **(f)**.

Metabolomics analysis was performed on all samples of the three groups, identifying a total of 676 known compounds. PCA was conducted on the metabolic data to assess group differentiation. The results showed that samples from the same group clustered together, while those from different groups were moderately separated (Figure 2a). Based on the classification information from the Human Metabolome Database (HMDB), the 676 identified metabolites were categorized into 15 classes. Among them, lipids and lipid-like molecules, organic acids and derivatives, and organoheterocylic compounds were the most abundant (Figure 2b). Differential analysis revealed 153, 71, and 57 DIMs between CAC and Control, CCS and Control, and CAC and CCS, respectively (Figures 2c,d). Subsequent KEGG enrichment analysis demonstrated that DIMs in CAC were mostly related to sphingolipid metabolism, linoleic acid metabolism, alanine, aspartic acid and glutamic acid metabolism, as well as citrate cycle pathways (Figure 2e). In contrast, DIMs in CCS were mainly enriched in pathways such as sphingolipid metabolism, glycerophospholipid metabolism, and arachidonic acid metabolism (Figure 2f).

**Figure 3:**
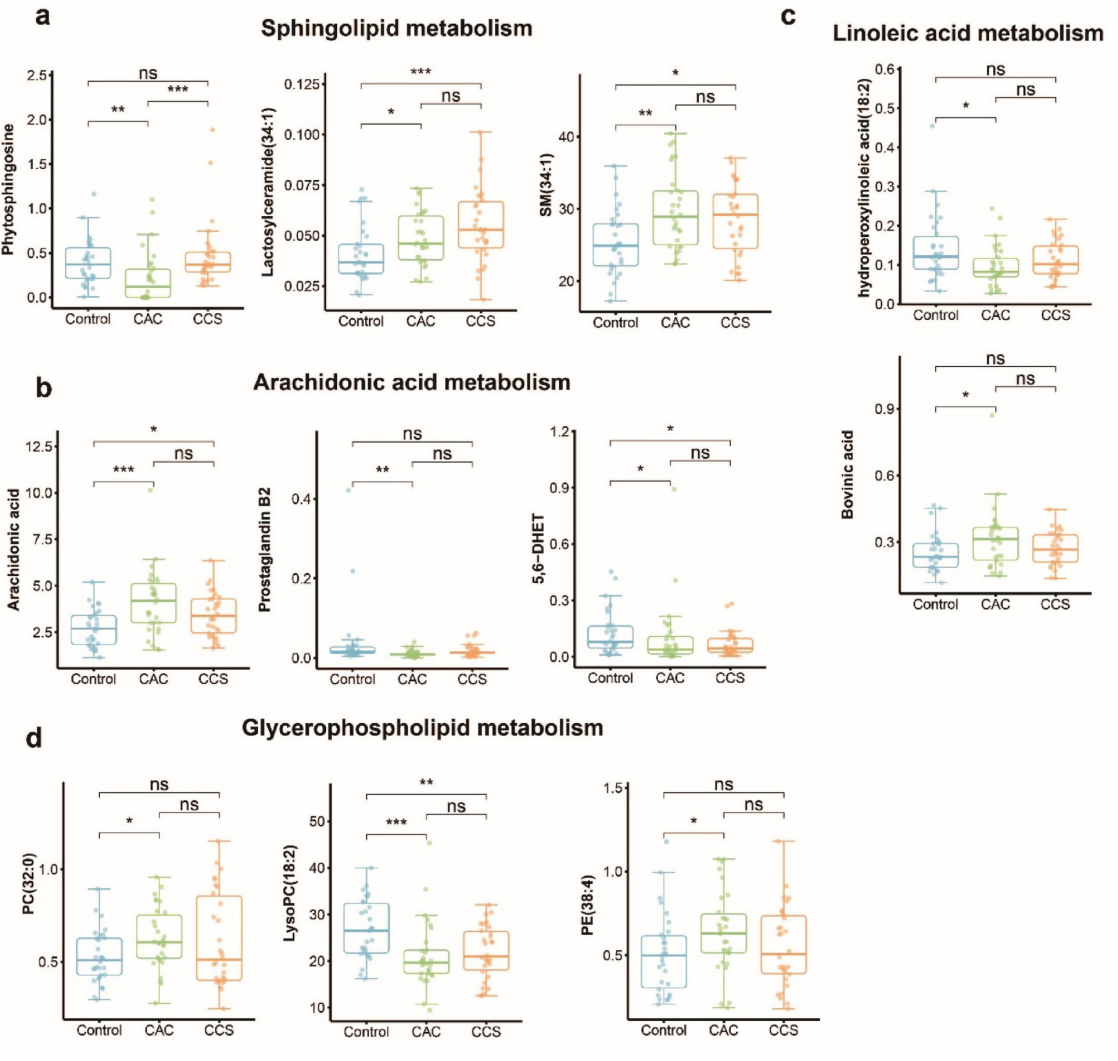
Relative abundance of metabolites in major KEGG pathways: sphingolipid metabolism **(a)**, arachidonic acid metabolism **(b)**, linoleic acid metabolism **(c)**, and glycerophospholipid metabolism **(d)** Differences between groups were assessed using the Mann–Whitney U test. Statistical significance is indicated as follows: * *p* < 0.05; ** *p* < 0.01; *** *p* < 0.001; ns, not significant.

Based on the KEGG enrichment results, we further examined the levels of metabolites identified within the enriched pathways among the three groups. In the sphingolipid metabolism pathway, phytosphingosine, lactosylceramide(34:1), and SM(34:1) showed significant differences across groups (Figure 3a). Within the arachidonic acid metabolism pathway, arachidonic acid and 5,6-DHET exhibited marked alterations in both the CAC and CCS groups compared with controls (Figure 3b). Furthermore, hydroperoxylinoleic acid(18:2) and bovinic acid in the linoleic acid metabolism pathway (Figure 3c), together with three metabolites in the glycerophospholipid metabolism pathway including PC(32:0), LysoPC(18:2), and PE(38:4) (Figure 3d), showed significant differences between the CAC and Control groups.

### 3.3 WGCNA and module-based co-expression network analysis of metabolites

**Figure 4:**
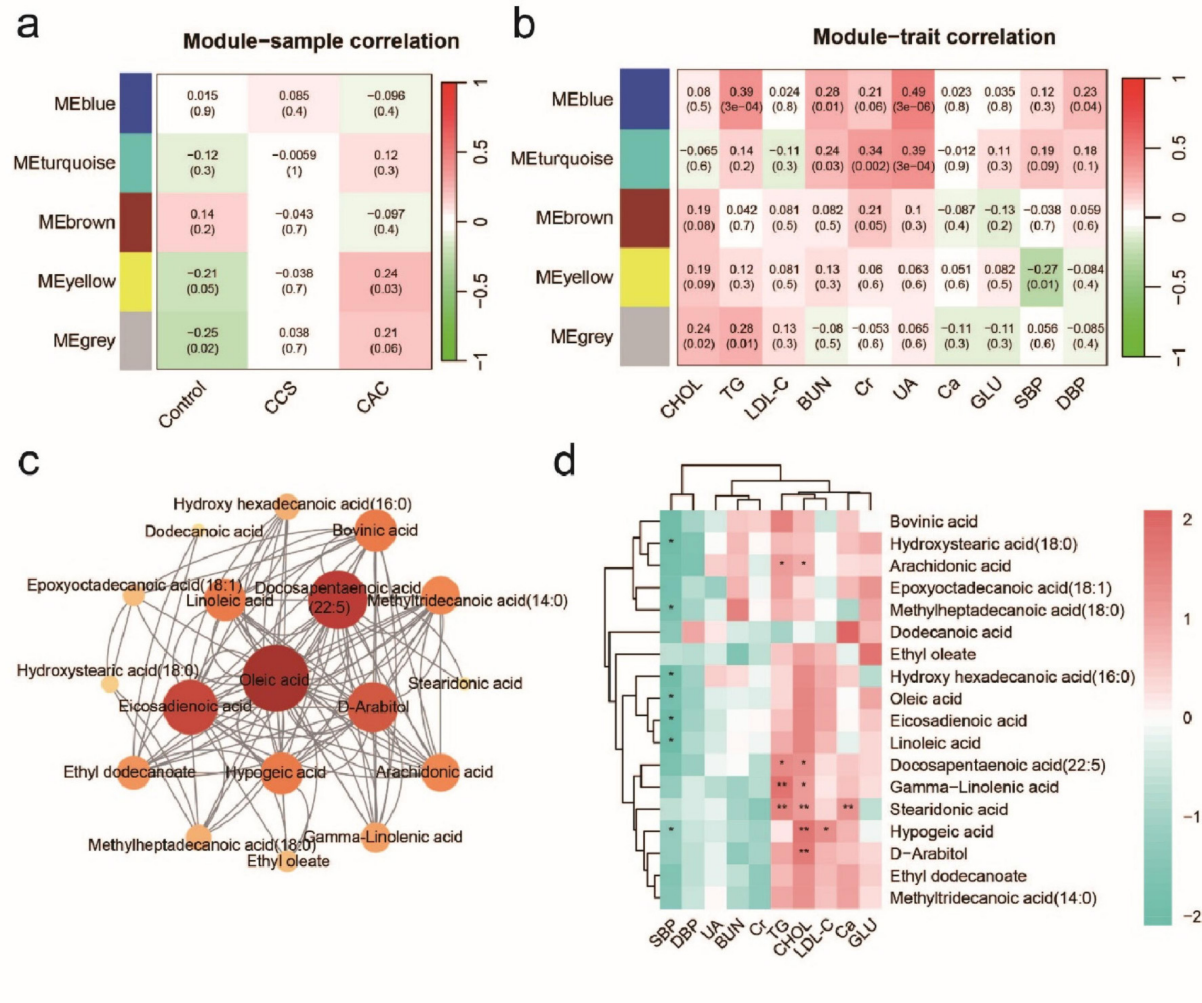
Weighted gene co-expression network analysis **(**WGCNA) and metabolite interaction network. **(a)** Module–sample group correlation heatmap from WGCNA. **(b)** Module–clinical parameter correlation heatmap from WGCNA. The color scales indicate the correlation coefficient, with red for positive and green for negative correlations. Numbers in parentheses are the corresponding *p*-values. **(c)** Co-expression network displaying core metabolites within the yellow module. Nodes represent metabolites, and edges represent significant co-expression relationships. The size and color intensity of nodes reflect the centrality and connectivity of metabolites in the network. **(d)** Heatmap showing correlations between core metabolites and clinical parameters. The color scales indicate the correlation coefficient, with red for positive and cyan for negative correlations. Asterisks denote statistical significance: * *p* < 0.05, ** *p* < 0.01.

WGCNA was performed on all detected metabolites, resulting in five co-expression modules. The module–trait correlation heatmap showed that the yellow module was significantly positively correlated with CAC (r = 0.24, *p* = 0.03), and was therefore selected for further analysis (Figure 4a). Additionally, the module–trait correlation heatmap revealed that the blue module was significantly negatively correlated with SBP (r = −0.27, *p* = 0.01) (Figure 4b). Correlation analysis was performed on metabolites in the yellow module, and those with r > 0.65 and *p* < 0.05 were selected to construct a co-expression network. Among them, metabolites with an outdegree > 10 were defined as core metabolites and visualized in the network, such as oleic acid and eicosadienoic acid (Figure 4c). Subsequent correlation analysis between these metabolites and clinical parameters showed that they were negatively correlated with SBP, but positively correlated with TG and CHOL (Figure 4d).

### 3.4 Metabolite correlation analysis and MCODE identified metabolite subnetworks associated with differences between the CAC and CCS

To further investigate the correlations among DIMs, Pearson correlation analysis was performed on DIMs between the Control and CAC groups, as well as between CCS and Control groups. These correlations were used to construct interaction networks. The molecular complex detection (MCODE) algorithm was then applied to calculate a score for each metabolite node within the networks. The top two nodes with the highest MCODE scores were selected as core nodes for further clustering, resulting in two prominent sub-networks associated with CAC and CCS.

**Figure 5:**
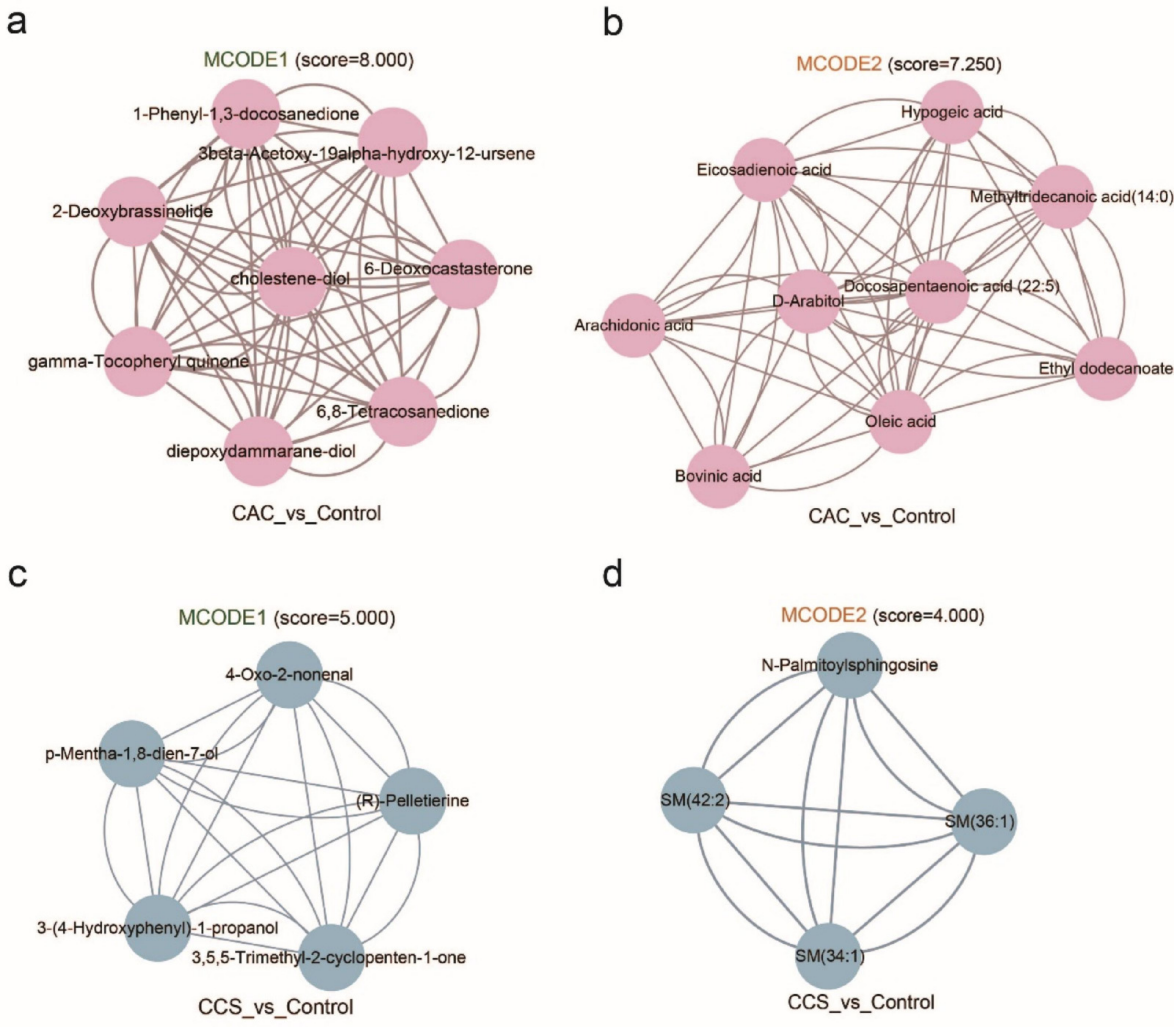
Metabolite correlation analysis and MCODE identified metabolite subnetworks associated with differences between the CAC and CCS groups. **(a,b)** Key sub-networks identified between CAC and Control; **(c,d)** Key sub-networks identified between CCS and Control.

In the comparison between the CAC and Control groups, metabolites such as oleic acid, methyltridecanoic acid (14:0), and arachidonic acid were identified as key nodes within the two sub-networks (Figures 5a,b**)**. Similarly, two sub-networks were identified in the comparison between CCS patients and the Control group, with metabolites such as N-palmitoylsphingosine and SM(42:2) appearing as prominent nodes (Figures 5c,d). These results highlight the central metabolites and sub-network structures associated with CAC and CCS, suggesting their potential involvement in the metabolic architecture underlying these conditions.

### 3.5 Random forest analysis of metabolites contributing to group differentiation

**Figure 6:**
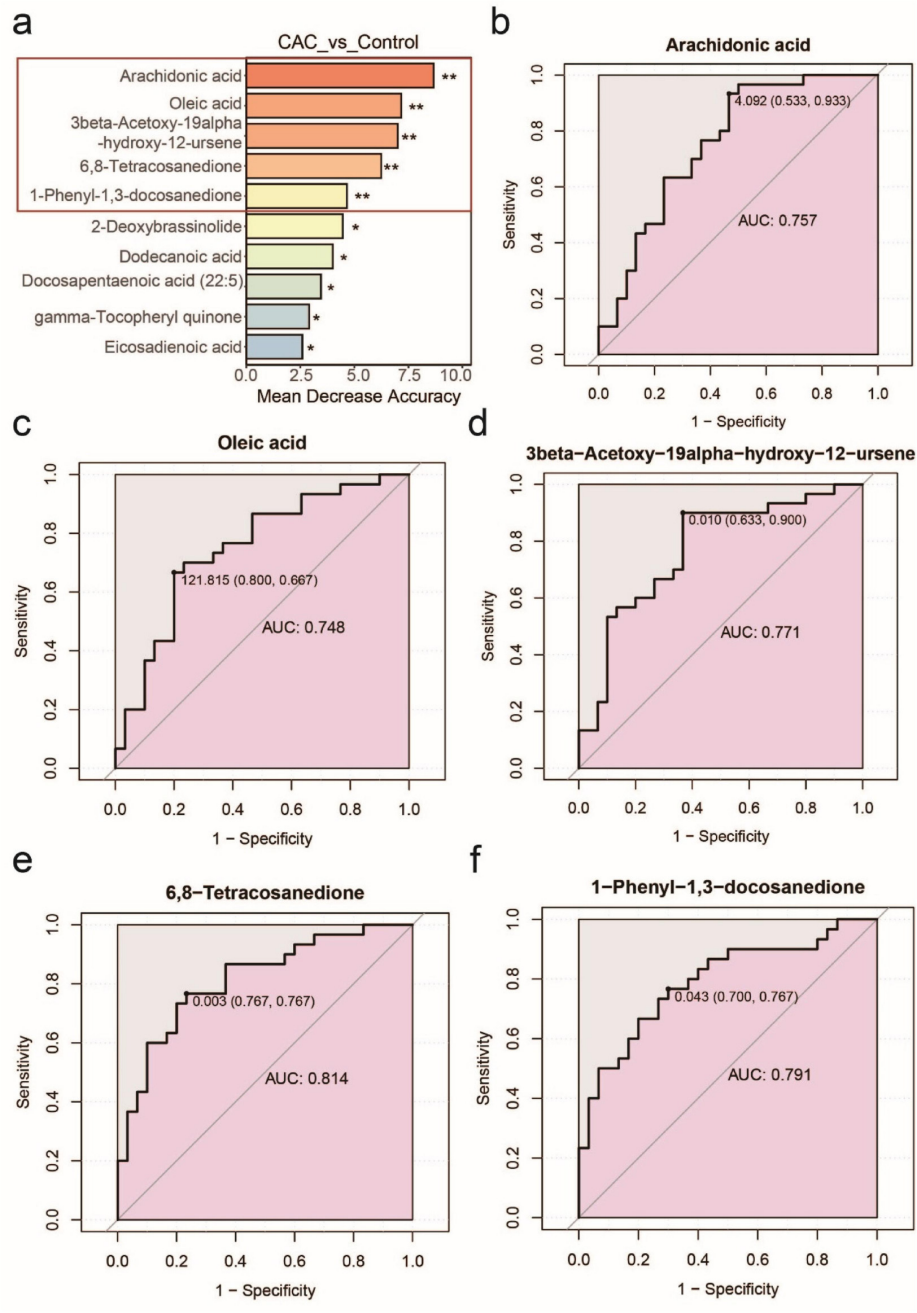
Random forest analysis and receiver operating characteristic (ROC) curves were used to explore the relative importance of metabolites potentially associated with group differences in CAC. **(a)** Random forest model constructed using metabolites from the yellow module associated with CAC, combined with metabolites from the CAC MCODE sub-networks. **(b–f)** ROC curves showing the discriminatory performance of selected metabolites.

To further explore metabolite patterns identified by WGCNA, we applied random forest analysis (Figure 6a) to the 18 metabolites from the yellow module to evaluate their relative importance in distinguishing the study groups (Figure 4c), along with additional metabolites from the MCODE sub-networks of DIMs between CAC patients and controls (Figures 5a,b). Random forest analysis suggested that several metabolites showed discriminatory performance, including arachidonic acid (AUC=0.757), oleic acid (AUC=0.748), 1-Phenyl-1,3-docosanedione (AUC=0.791), 6,8-Tetracosanedione (AUC=0.814), and 3beta-Acetoxy-19alpha-hydroxy-12-ursene (AUC=0.771). Among them, arachidonic acid and oleic acid were the most important contributors to group classification (Figures 6b–f), suggesting that these fatty acids may be associated with metabolic differences observed between the groups.

**Figure 7:**
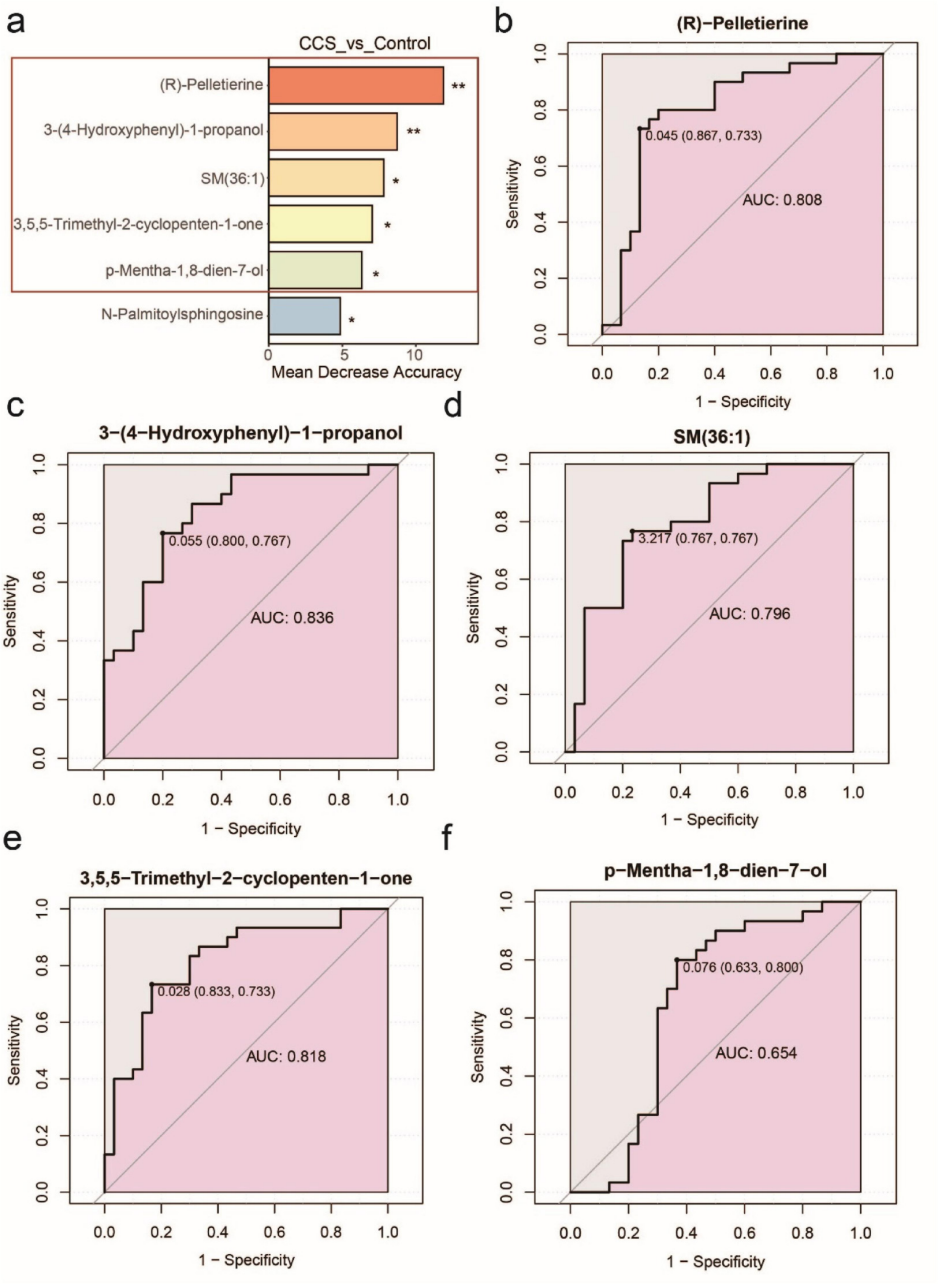
Random forest analysis and ROC curves were used to explore the relative importance of metabolites potentially associated with CCS. **(a)** Random forest model constructed using metabolites from the CCS MCODE sub-networks. **(b–f)** ROC curves showing the discriminatory performance of selected metabolites.

Similarly, we constructed a random forest model using the nine metabolites from the MCODE subnetwork to explore their contribution to distinguishing the CCS group (Figure 7a). Several metabolites showed relatively higher AUC values, including (R)-pelletierine (AUC = 0.808), 3-(4-hydroxyphenyl)-1-propanol (AUC = 0.836), and SM(36:1) (AUC = 0.796) (Figures 7b–f**)**.

### 3.6 Metabolite clustering and pathway enrichment analysis

**Figure 8:**
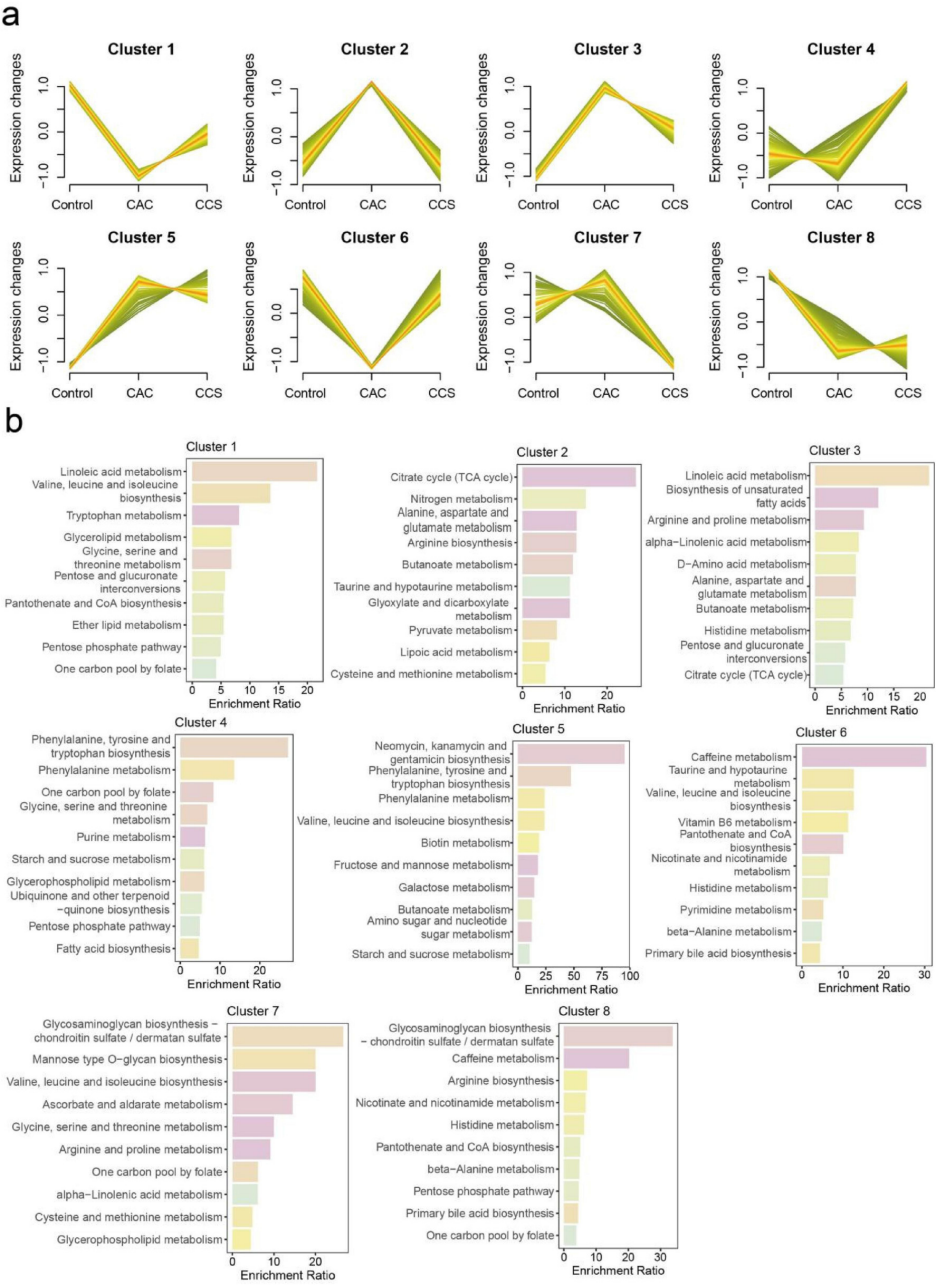
**(a)** metabolite clustering analysis of all metabolites. **(b)** Functional enrichment analysis of all clusters.

K-means clustering analysis was performed on the entire metabolite dataset to gain a comprehensive understanding of plasma metabolite changes in CAC and CCS patients. A total of eight clusters were identified (Figure 8a). Notably, Cluster4 and Cluster6 exhibited the highest metabolite abundance in CCS, while their levels were lowest in CAC. These metabolites were mainly concentrated in pathways such as taurine and hypotaurine metabolism, caffeine metabolism, phenylalanine, tyrosine and tryptophan biosynthesis, and phenylalanine metabolism. Metabolites in Cluster2, Cluster3, Cluster5 and Cluster7 had the highest abundance in CAC, which was significantly higher than that in Control and CCS. These metabolites were mainly enriched in the biosynthesis of neomycin, kanamycin and gentamicin, linoleic acid metabolism, citrate cycle (TCA cycle), butyric acid metabolism, valine, leucine and isoleucine biosynthesis, D-amino acid metabolism, and other pathways. Clusters 1 and 8 showed the highest metabolite abundance in the Control group, with levels significantly lower in both CCS and CAC patients. These clusters reflected the metabolites that were more prevalent under normal conditions but decreased as the disease progressed. Specifically, the pathways enriched in these clusters included linoleic acid metabolism, valine, leucine and isoleucine biosynthesis, glycosaminoglycan biosynthesis, chondroitin sulfate/dermatan sulfate, as well as other critical metabolic processes (Figure 8b).

## 4 Discussion

Coronary artery calcification (CAC) and chronic coronary syndrome (CCS) represent important manifestations of coronary atherosclerotic disease and are closely associated with metabolic dysregulation (27, 28). In the present study, we performed a comprehensive metabolomic analysis integrating DIM screening, network analysis, and machine learning approaches to explore metabolic alterations associated with CAC and CCS. Our findings suggest substantial perturbations in lipid metabolism, particularly in sphingolipid, glycerophospholipid, and polyunsaturated fatty acid metabolism, and highlight several metabolites that may contribute to the pathophysiology of CAC and CCS. Baseline analysis showed no significant differences among groups in most clinical parameters, indicating that the CAC, CCS, and control groups were generally comparable. This minimizes potential confounding effects and suggests that the observed metabolic alterations are more likely related to disease status rather than baseline demographic or clinical differences.

Functional enrichment analysis revealed that plasma metabolites in CAC patients were enriched in pathways such as alanine, aspartic acid, glutamate metabolism, valine, leucine, and isoleucine biosynthesis. This finding is consistent with previous studies indicating that amino acid metabolism is often disrupted in patients with cardiovascular diseases. Shah et al. (29) reported a significant correlation between plasma branched-chain amino acids and the incidence of coronary artery disease and myocardial infarction. Yi et al. (30) identified potential biomarkers in the plasma of patients with coronary heart disease, including valine, tyrosine, alanine, and glutamine. Furthermore, studies have shown that the catabolism of valine and isoleucine can provide energy, promote protein synthesis in the myocardium, reduce myocardial stress injury, and facilitate the repair of vascular smooth muscle cells (31, 32). These metabolic alterations may therefore represent a compensatory or protective response to cardiovascular stress. In CAC patients, abnormal metabolic pathways also included the TCA cycle, consistent with a previous metabolomic analysis of blood samples from patients with coronary heart disease (33). These findings suggest that disruptions in both amino acid metabolic pathways (34) and energy metabolism (35, 36) may be key contributors to the development of CAC. In CCS patients, metabolites were primarily enriched in arachidonic acid metabolism, taurine and hypotaurine metabolism, phenylalanine, tyrosine and tryptophan biosynthesis, as well as phenylalanine metabolism. Notably, Liu et al. (2) reported that taurine and hypotaurine metabolism was negatively correlated with the severity of coronary heart disease. Moreover, increased activity of phenylalanine, tyrosine, and tryptophan biosynthesis pathways may enhance myocardial contractility, reduce oxidative stress, and modulate energy metabolism (37, 38).

Further comparison between CAC and the Control groups revealed that the DIMs were significantly enriched in sphingolipid metabolism and linoleic acid metabolism pathways. The ASM/ceramide pathway of sphingolipid metabolism can promote low-density lipoprotein aggregation and macrophage foam cell formation, thereby accelerating the progression of atherosclerosis (39, 40). In addition, sphingomyelin promotes the proliferation of vascular smooth muscle cells, and its metabolite, ceramide, has been shown to correlate positively with the severity of coronary artery disease (41, 42). In the present study, several metabolites in the sphingolipid metabolism pathway—lactosylceramide(34:1) and SM(34:1)—were significantly elevated in both the CAC and CCS groups, reflecting potential alterations in sphingolipid metabolism that warrant further investigation in independent cohorts. Linoleic acid derivatives are associated with oxidative stress and lipid peroxidation, processes known to accelerate endothelial injury and plaque instability. Increased oxidative metabolites of linoleic acid may therefore reflect enhanced oxidative stress in patients with coronary calcification (43, 44). In this study, using a random forest model, we investigated metabolites potentially associated with differences between CAC patients and healthy individuals, including arachidonic acid and oleic acid. Oleic acid has been found to be linked to vascular calcification and can be modulated through n-3 fatty acid supplementation (45). Arachidonic acid is a critical precursor of various eicosanoids, including prostaglandins, leukotrienes, and epoxyeicosatrienoic acids, which play major roles in inflammation, vascular tone regulation, and thrombosis (46). Dysregulation of arachidonic acid metabolism has been widely implicated in cardiovascular diseases. Elevated levels of arachidonic acid may promote inflammatory signaling and vascular remodeling, thereby contributing to atherosclerosis progression and coronary calcification (47, 48). Collectively, these findings suggest that lipid metabolic changes may reflect group-associated differences in coronary artery disease.

Another important metabolic pathway identified in this study is glycerophospholipid metabolism. Metabolites such as lysophosphatidylcholine (LysoPC), phosphatidylcholine (PC), and phosphatidylethanolamine (PE), were significantly changed, reflecting disturbances in membrane composition, lipoprotein metabolism, and inflammatory signaling (49–51). In particular, LysoPC has been reported to promote endothelial dysfunction, vascular inflammation, and monocyte recruitment during atherosclerosis development (52–54). To further explore the relationships between metabolic features and clinical parameters, correlation analysis was performed. Several metabolites showed negative correlations with SBP and positive correlations with triglyceride and cholesterol levels, indicating that these metabolic alterations may be associated with differences in cardiometabolic profiles between the study groups.

The present study demonstrates that CAC and CCS are characterized by coordinated disturbances in lipid metabolism, fatty acid oxidation, and inflammatory lipid mediator pathways. Sphingolipids, phospholipids, and polyunsaturated fatty acids constitute interconnected metabolic networks that may contribute to vascular inflammation, endothelial dysfunction, and coronary calcification. Metabolites including oleic acid, arachidonic acid, and sphingomyelin, showed consistent alterations, suggesting they could provide insights into metabolic changes associated with coronary artery disease. These findings highlight the potential relevance of lipid metabolic pathways in disease progression, although further studies with larger cohorts are needed to validate these observations.

This study has several limitations. First, the sample size was relatively small, which limits statistical power, particularly for high-dimensional analyses such as WGCNA, MCODE, and random forest modeling. Second, the study was conducted in a single cohort without a fully independent external validation dataset due to the limited accessibility of publicly available metabolomics data with corresponding CAC information. Future studies with larger, multi-center cohorts are warranted to confirm and generalize these findings. Thirdly, it is noteworthy that some metabolites identified in this study, including (R)-pelletierine, 2-deoxybrassinolide, and 3β-acetoxy-19α-hydroxy-12-ursene, among others, are not commonly reported in human plasma and may originate from dietary intake or environmental exposure, rather than solely reflecting alterations in endogenous metabolic pathways. Therefore, the observed intergroup differences in these metabolites may partially reflect variations in lifestyle or dietary patterns, instead of directly contributing to the pathological processes of CCS or CAC. Future studies incorporating detailed dietary assessments and targeted quantitative validation are warranted to clarify the sources of these metabolites. In the CAC group, creatinine, blood urea nitrogen, and fasting glucose were higher than in controls. Renal dysfunction and coronary calcification are closely interrelated, potentially bidirectional processes, reflecting shared systemic vascular pathology (55, 56). Elevated glucose levels further reflect underlying metabolic disturbances commonly observed in patients with vascular calcification (57). These differences may represent interrelated manifestations of a shared pathophysiological continuum rather than an unintended imbalance in cohort selection. Nevertheless, these factors may partially influence metabolite profiles and should be considered when interpreting the metabolomics results. Finally, while this study identified metabolites associated with CAC and CCS based on statistical and bioinformatics analyses, no experimental validation was performed to confirm their functional roles. In addition, due to the untargeted lipidomics strategy used, some lipid isomers may not have been fully resolved at the structural level. Therefore, these findings should be considered preliminary and exploratory. However, the observed metabolic alterations provide potential insights into pathways that may be involved in coronary artery disease, which could inform future mechanistic studies and hypothesis-driven investigations.

## Data Availability

The raw data supporting the conclusions of this article will be made available by the authors, without undue reservation.
